# Angle-Dependent Adhesive Mechanics in Hard–Soft Cylindrical Material Interfaces

**DOI:** 10.3390/ma18020375

**Published:** 2025-01-15

**Authors:** Thao H. Pham, Iakov A. Lyashenko, Valentin L. Popov

**Affiliations:** 1 Department of System Dynamics and Friction Physics, Institute of Mechanics, Technische Universität Berlin, 10623 Berlin, Germany; pham.19@campus.tu-berlin.de (T.H.P.); v.popov@tu-berlin.de (V.L.P.); 2 Department of Theoretical and Applied Mechanics, Samarkand State University, Samarkand 140104, Uzbekistan; 3 Center of Advanced Studies in Mechanics, Tribology, Bio- and Nanotechnologies, Samarkand State University, Samarkand 140104, Uzbekistan

**Keywords:** adhesion, hertzian contact, elliptical contact, contact area, indentation, elastomer

## Abstract

In this research, the adhesive contact between a hard steel and a soft elastomer cylinder was experimentally studied. In the experiment, the hard cylinder was indented into the soft one, after which the two cylinders were separated. The contact area between the cylinders was elliptical in shape, and the eccentricity of this increased as the angle between the axes of the contacting cylinders decreased. Additionally, the adhesive pull-off force and the contact area increased with a decrease in the angle between the cylinders. The use of a transparent elastomer allowed for observation of the shape of the contact in real time, which facilitated the creation of videos demonstrating the complete process of contact failure and the evolution of the ellipse shape, depending on the distance between the cylinders and normal force. These findings contribute to a better understanding of adhesive interactions in elliptical contacts between cylinders and can be applied to fields such as soft robotics, material design, and bioengineering, where precise control over adhesion and contact mechanics is crucial.

## 1. Introduction

The rapid advancement of technology necessitates the miniaturization of components and the investigation of their contact behavior at that small scale [1,2]. Contact behavior is influenced by several factors, including the contact geometry, external forces, internal adhesion, and the intrinsic properties of the material [3,4]. It is crucial to acknowledge that the influence of adhesion intensifies when the contact body diminishes in size or exhibits reduced stiffness [3]. In contrast, the adhesive properties are influenced by surface roughness, contamination, and wear [5]. Such contact behavior, in which the adhesive force is of significant influence, is commonly observed in MEMS (microelectromechanical systems) [1], bioadhesives [6,7], smart surfaces [8,9], smart coatings [10], robotics [11], and more. Given the pivotal role of adhesive attraction in determining contact behavior, it is essential to investigate the parameters that may influence adhesive forces.

The initial theoretical considerations of the contact problem were established by Hertz in 1881, wherein he derived a relationship between the contact force and the displacement of two elastic and curved surfaces in contact [12]. It should be noted that the influence of adhesion was not taken into account in his theory. Despite Hertz deriving the relations for general curved surfaces, which result in elliptical contacts, many subsequent contact theories were based on contacting spheres or a sphere in contact with a plane, which result in theoretically round contact areas. In 1975, Johnson, Kendall, and Roberts extended Hertz’s theory by considering adhesion in the limiting case of contacting elastic spheres. This theory, which is widely accepted in the field, is known as the JKR theory [13].

It is important to note that in many applications, the contacting bodies are not perfectly spherical. This makes the circular contact a specialized case. Moreover, elliptical contacts are far more common, as the shape can be achieved through the contact of elliptical bodies or two cylindrical rods rotated at an angle relative to one another. In applications, elliptical contact areas are observed in bearings [14], cylindrical gears [15] or between polymer fibers in fibrous structures [16]. Cylindrical contact geometry plays a crucial role in the manufacturing of rubber and plastic products, where adhesive interactions during molding and pressing processes must be carefully considered [17]. In welding and bonding, particularly in the automotive and aerospace industries, cylindrical contact is essential for ensuring strong connections through proper adhesion [18]. In medicine, the development of implants often involves cylindrical contact [19], where understanding adhesive interactions is vital for ensuring the safety and longevity of the devices [20]. Understanding the adhesion contact between cylindrical objects is crucial for elucidating the mechanism of cell infection by viruses [21]. In optics and microscopy, cylindrical contact between components affects the quality of the image, as adhesive forces can influence performance [22]. Finally, in manufacturing technologies such as stamping [23] and forging [24], where cylindrical contacts are commonly encountered, adhesion between materials and tools significantly impacts the precision and efficiency of the processes. Adhesive cylindrical contacts are also common in modern applications, such as soft robotics [25,26].

In the 1990s, Greenwood conducted an analysis of the elliptical Hertzian contact, deriving approximating methods to determine the contact area, contact pressure, and deformation. These methods were based on the assumption of circular contact relations with an effective radius, which allowed for simplifications in the calculations [27]. The extension of the adhesive circular contact problem to the elliptical contact problem with the JKR Model was attempted by Johnson and Greenwood in 2005 [28,29]. In the Hertzian theory, the elliptical contact area is a result of the contact between curved surfaces [12,28]. In the theoretical framework proposed by Johnson and Greenwood, it is assumed that in the absence of adhesion, the contact between the two surfaces assumes an elliptical shape [29]. In light of the aforementioned considerations regarding adhesion, it remains unclear whether the contact area retains its elliptical shape, given that it is significantly influenced by surface energy. The theory demonstrated that the contact maintains an elliptical shape, yet the eccentricity varies continuously in accordance with the applied load. This stands in contrast to Hertz’s theory, wherein the eccentricity remains constant across the entire range of normal forces. Moreover, the eccentricity is consistently less than that predicted by Hertz in the absence of adhesion. Another pivotal finding is that during the detachment phase, the initially elliptical contact area transitions towards a circular shape as the normal force approaches the pull-off force. The change in shape affects the maximal adhesion force, for example, the pull-off force. The magnitude of the pull force is contingent upon the ratio between the relative radii of curvature. For ratios that are higher, the pull-off force decreases [28,30].

In addition to the analytical calculations of the elliptical contact, numerical calculations were conducted [31,32]. One such calculation extended the double-Hertz model, in which adhesion is calculated numerically by subtracting the Hertzian pressure distributions [31]. In this instance, the adhesive contact region is the result of two non-equivalent Hertzian contact areas. The approximate JKR model for elliptical contacts is based on the assumption that the stress intensity values at the tip of both the minor and major axes are identical [29,31].

Building upon this theoretical framework, the present experimental study aimed to investigate the contact behavior of cylindrical rods in contact with each other, separated by an elastomeric sheet. Specifically, we examined how the contact angle affects the adhesive force and contact area for three rod pairings with varying radii. This study expands on prior research [28] by utilizing larger cylinders, offering new insights into the contact mechanics of elliptical adhesive interactions. The advantage of our work is the inclusion of Supplementary Videos, which provide a detailed view of the indentation and contact failure processes for various angles between the contacting cylinders.

## 2. Materials and Methods

The experimental setup comprised a steel cylinder attached to a three-axis force sensor ME K3D40 (ME-Meßsysteme, Henningsdorf, Germany). The signal was amplified using an analog amplifier GSV-1A4 SubD37/2 (ME-Meßsysteme, Henningsdorf, Germany) and then transmitted to a 16-bit Multifunction I/O Device NI USB-6211 (National Instruments, Austin, TX, USA), which was then connected to a computer. The vertical movement of the indenter was facilitated by a linear stage PI M-403.2DG (Physik Instrumente (PI) GmbH & Co. KG, Karlsruhe, Germany), which was operated by a controller PI C-863 (Physik Instrumente (PI) GmbH & Co. KG, Karlsruhe, Germany). A program was developed in LabVIEW Full Development System ver. 2019 (National Instruments, Austin, TX, USA) for the purpose of controlling the equipment and collecting the data.

The counterbody was a thermoplastic polystyrene-type gel sheet, TANAC CRG-N0505 (TANAC Co., Ltd., Gifu, Japan). It was placed on a half-cylinder mounted on a manually rotating stage PI M-037.00 (Physik Instrumente (PI) GmbH & Co. KG, Karlsruhe, Germany), which allowed for easy and precise adjustment of the angle between the axes of the contacting cylinders. The rotation stage was located on a PI M-044.00 tilt stage (Physik Instrumente (PI) GmbH & Co. KG, Karlsruhe, Germany), at the center of which a hole with a diameter of 45 mm was made to allow observation of the contact area with the camera from below the contact point. The tilt stage allowed the angle of the elastomeric surface (counterbody) to be changed independently in two horizontal directions.

**Figure 1:**
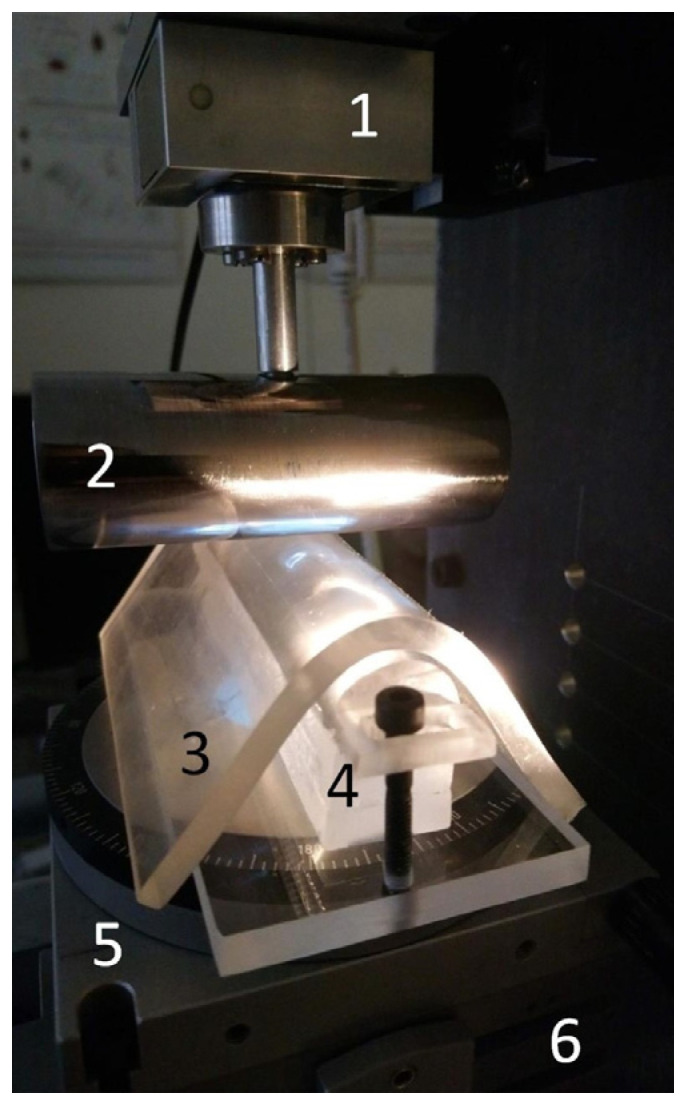
The picture of experimental setup in the contact zone: 1—three-axis force sensor ME K3D40; 2—polished steel cylindrical indenter with radius $R_{up}=15$ mm; 3—TANAC CRG N0505 elastomer sheet (100 mm × 100 mm and thickness of 5 mm); 4—half-cylinder made of acryl glass; 5—PI M-037.00 rotation stage, which allows setting any angle between the upper cylinder and the cylinder at the bottom; 6—PI M-044.00 tilt stage.

Both the bottom half-cylinder and the elastomer CRG-N0505 sheet were transparent, allowing the camera, positioned beneath the apparatus, to capture the contact area. It should be noted that the lower part remained stationary throughout the experiment; only the steel indenter was lowered into the elastic sheet and then withdrawn from the contact area. The same steel indenter, with a radius of $R_{up}=15\;$mm, was utilized in all experimental series. However, various counter half-cylinders were utilized, differing in size and material. The experimental setup is depicted in Figure 1.

**Figure 2:**
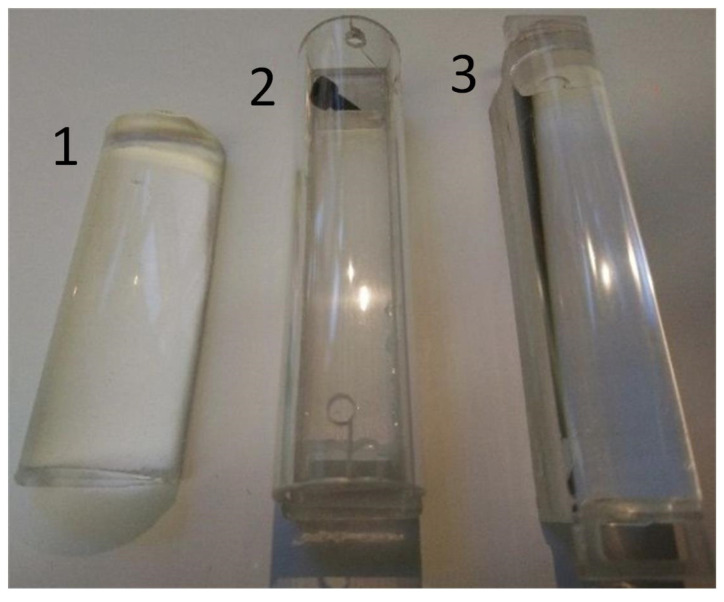
The image depicts three half-cylinders, which were utilized as a substrate beneath the elastomer layer to alter the layer’s curvature. (1) A rubber half-cylinder made of “TFC Silikon Kauchuk Typ 19” with $R_{1}^{+}\approx 15$ mm; (2) half of a hollow acrylic glass tube with $R_{2}^{+}\approx 12.5$ mm; (3) a fully filled acrylic half cylinder with $R_{3}^{+}\approx 10$ mm.

Three sets of experiments were conducted with different bottom half-cylinders. The first half-cylinder was manufactured using a two-component liquid rubber material “TFC Silikon Kauchuk Typ 19” (TFC Troll Factory GmbH, Walsrode, Germany). Upon solidification, the material exhibited a radius of $R_{1}^{+}=15$ mm. In contrast to the first half-cylinder, the second half-cylinder, constructed from an empty acrylic glass tube with an outer radius of $R_{2}^{+}=12.5\;$mm, was hollow and not completely filled. The last cylinder was constructed from fully filled acrylic glass with the smallest radius of $R_{3}^{+}=10\;$mm. All substrates are illustrated in Figure 2. The elastomer sheet utilized in this experiment was a perfectly transparent material, CRG-N0505, which exhibited robust adhesive properties (a high value of specific work of adhesion $\gamma_{12}$ in the elastomer–steel contact zone). The same elastomeric sheet was places on all half-cylinders and had a thickness of h=5 mm. This resulted in the bottom cylinder radius in the contact zone being approximately 20 mm, 17.5 mm and 15 mm, respectively, for $R_{1},$ $R_{2}$ and $R_{3}$. Consequently, the radius of the upper and lower cylinders for the last experimental series was identical.

In all experiments, a steel indenter was pressed into a soft layer of CRG N0505 elastomer, which was positioned on a cylinder made of a stiffer elastomer or acrylic glass. The elastic modulus of steel is about $2\times 10^{5}$ MPa, the elastic modulus of acrylic glass is about $3\times 10^{3}$ MPa, and the elastic modulus of the studied elastomer is approximately 0.054 MPa. This means that in all cases under consideration, the elastic modulus of the cylinders exhibited markedly higher values in comparison with the elastic modulus of the elastomer material. It can thus be concluded that the observed deformation is confined to the upper layer of the elastomer. Given the notable thickness of the elastomer (h=5 mm), it is not accurate to assume that the radius of the lower cylinder in the contact is equal to the sum of the radius of the substrate and the elastomer thickness. This is due to the fact that the deformation of the elastomer at the top and bottom levels is not identical. At the top level, tensile strains are present, while at the bottom level, compression strains are observed. Thus, the total radius can only be approximated with the use of the “≈” symbol, as demonstrated for the last cylinder $R_{3}\approx 15\;\mathrm{mm}$. One potential method for mitigating this phenomenon is to utilize elastomers with exceedingly thin thicknesses h. However, in such instances, the underlying half-cylinders may undergo considerable deformation, resulting in a shift away from the half-space approximation.

A series of experiments were conducted on each cylindrical substrate, following a uniform procedure as will be described in detail below. Following initial contact, the steel cylinder was indented to a maximum depth of $d_{max}=0.3$ mm at a constant velocity of v=5 µm/s. Once the maximal indentation depth was reached, the indenter was withdrawn from the elastomeric sheet at a significantly slower velocity of v=0.2 µm/s up to the moment of full detachment. For adhesive contact, complete detachment and contact breakage occur at negative indentation depths d<0 mm, whereas positive indentation depths are in an interval of $0<d<d_{max}$, where d=0 mm denotes the initial contact point between the surfaces. Photographs of the contact area were taken at regular intervals throughout the process. Following the completion of a full indentation and detachment cycle, the lower portion of the contact area was rotated by 10° and the entire process was then repeated. In the initial cycle, the angle between the axes of the upper and lower contact bodies was 90°. This angle was then gradually reduced by 10° until it reached 10°. Furthermore, an additional experiment was conducted at an angle of 15° to allow for the performance of 10 experiments for each contact pairing. Angles of less than 10° were not employed, as the resulting contact area exceeded that of the contact frame at this angle.

This study investigated quasi-static adhesive contact. It is considered quasi-static in the sense that indentation and detachment were performed at low indenter velocities, where viscoelastic effects could be neglected. The contacting surfaces were smooth to avoid the influence of surface roughness. If necessary, the described experimental method can be used to study the effects of viscoelasticity, roughness, or surface energy in contact between different materials. In our recent work [33], we explored all the aforementioned cases. Specifically, it was experimentally demonstrated that an increase in the specific adhesion energy and the detachment velocity of the indenter from the elastomer leads to a monotonic increase in adhesive contact strength. However, the effect of roughness is less straightforward: as the amplitude of surface roughness increases, the adhesive contact strength initially increases and then decreases again. It should be noted, however, that in [33], the common case of contact between a soft elastomer and a rigid spherical indenter was studied, and in adhesive contacts, contact geometry plays a significant role. Therefore, the results of [33] cannot be directly generalized to the data obtained in this study, and additional research is required to clarify the influence of the mentioned effects for elliptical contacts.

## 3. Results

**Figure 3:**
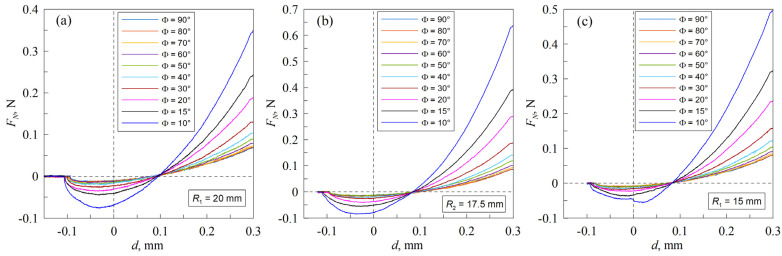
Experimental dependencies of the normal force $F_{N}$ vs. indentation depth d for different contact configurations, where $R_{1}\approx 20$ mm (**a**), $R_{2}\approx 17.5\;$mm (**b**), and $R_{3}\approx 15\;$mm (**c**). Each figure consists of 10 curves; different curves correspond to different values of the angle between cylinders (Φ=90°, 80°, 70°, 60°, 50°, 40°, 30°, 20°, 15° and 10°). There are Supplementary Videos for all figures (Video S1, Video S2, and Video S3).

Figure 3 presents the findings of the experiments conducted for all three investigated contact configurations. Each panel in the figure depicts ten dependencies, obtained at varying angles between the cylinders.

As evidenced by the Figure 3 and the Supplementary Videos, the normal force dependence on indentation depth exhibits a notable overlap at larger angles, particularly at Φ=90°, 80°, 70°. At such large angles, the complete contact area can be observed with the camera. At 90°, the shape of the contact area is approximately circular. As the angle of contact decreases, the contact area assumes an elliptical shape. As the cylinder is rotated further, the normal force dependence becomes more pronounced, with the normal force at the maximal indentation depth and the absolute value of the pull-off force (i.e., the force needed to break the contact between the two cylinders) exhibiting a notable increase. It is noteworthy that when examining solely the detachment phase, the dependencies for all angles converge at a similar point, which is at zero normal force and an indentation depth of approximately d≈0.1 mm.

While the maximal indentation depth $d_{max}$ was kept consistent across all contact configurations, Figure 3 illustrates that at the smallest angle of 10°, the maximal normal force first increases with the next smallest radius of the cylinder and then decreases again with the smallest radius, $R_{3}\approx 15\;$mm. Such behavior, at first glance, appears counterintuitive and should be explained in detail.

From previous experiments, the material characteristics of the utilized TANAC CRG N0505 elastomer sheet, including the Young’s modulus, the Poisson ratio, and specific work of adhesion, have been determined as follows: E≈54 kPa, ν≈0.47, and $\;\gamma_{12}\approx \;0.25\;J/m^{2}$ [34]. However, effective (actual) parameters can vary from one panel in Figure 3 to another, given that the lower substrate was manufactured in different ways for different cylinder radii.

**Figure 4:**
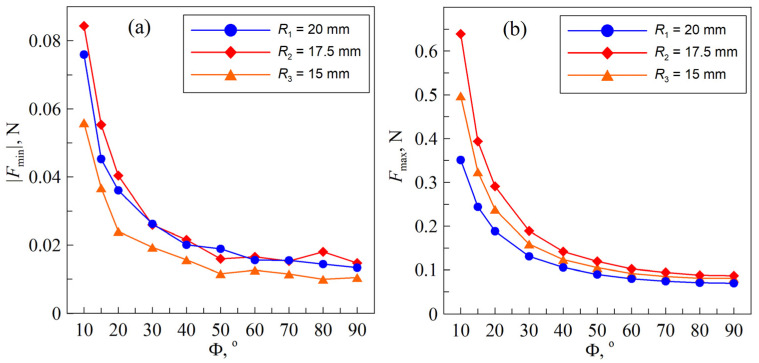
Dependencies of the “adhesive strength” of the contact (absolute value of adhesive pull-off force) $\left|F_{\min}\right|$ (**a**) and maximal normal force $F_{\max}$ (**b**) at maximal indentation depth $d_{max}=0.3\;$mm vs. angle Φ for data shown in Figure 3.

The use of different substrates beneath the elastomer may result in deviations in the normal force $F_{N}$ from those expected at the maximum indentation depth. This is because, as the external load increases, the measured reaction force may be caused not only by deformations in the upper layer of the soft elastomer but also by deformations in the underlying cylinders. However, if the external force and the contact radius are small, deformations will only occur in the elastomer layer (half-space limit). Therefore, the type of cylinder used should not affect the adhesive strength of the contact. Here, adhesive strength refers to the minimum external force required to completely break the adhesive contact, i.e., the pull-off force. This force corresponds to the minima of the dependencies shown in Figure 3. The dependencies of adhesive strength on the angle between the cylinders for all cases considered are shown in Figure 4a.

From this figure, it follows that the minimum adhesive strength is observed for the cylinder with the smallest radius $R_{3}\approx 15\;$mm, which is a correct and expected result. For the other two cases, the adhesive strength is approximately the same. However, it should be noted that the specific work of adhesion $\;\gamma_{12}$ strongly depends on temperature, humidity, surface chemical purity, and other parameters, which may vary across different experimental series.

Additionally, the JKR curves were calculated with the mentioned material characteristics and the combined radius of the two half-cylinders in the contact as

(1) $$R_{z}={\left(\frac{1}{R_{up}}+\frac{1}{R_{1,2,3}}\right)}^{-1}$$

where the radius of the upper steel cylinder was constant, $R_{up}=15\;$mm, in all experiments, and $R_{1,2,3}=20$, 17.5, and 15 mm in different experimental series. The JKR curves were calculated using the following standard relations [13]

(2) $$F_{N}\left(a\right)=\frac{4E^{*}}{3R_{z}}a^{3}-\sqrt{8\pi a^{3}E^{*}\gamma_{12}}$$

(3) $$d\left(a\right)=\frac{a^{2}}{R_{z}}-\sqrt{\frac{2\pi a\gamma_{12}}{E^{*}}}$$

where a is the contact radius. The reduced elastic modulus for the contact between a rigid cylinder and a soft elastomer is given by $E^{*}=E/\left(1-\nu^{2}\right)$, where E, ν and $\;\gamma_{12}$ are the parameters of the elastomer.

**Figure 5:**
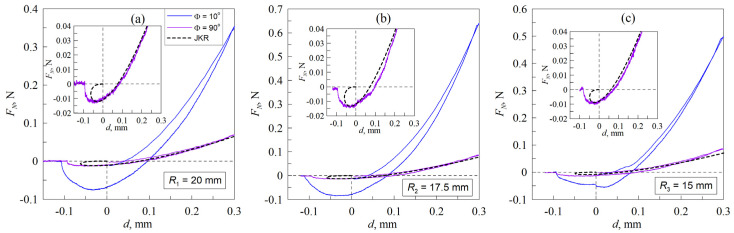
Experimental dependencies of the normal force $F_{N}$ vs. indentation depth d for different contact configurations, where $R_{1}\approx 20\;\mathrm{mm}$ (**a**), $R_{2}\approx 17.5\;\mathrm{mm}$ (**b**), and $R_{3}\approx 15\;\mathrm{mm}$ (**c**). The curves for the angles $\Phi =90^{o}$ and $\Phi =10^{o}$ are given (solid lines) with the analytically calculated JKR curves for spheres (dashed lines).

As can be seen from Figure 5, the experimental curves for the cases of the angle Φ=90° (circular contacts) show a similar behavior to the theoretical JKR curves, which are depicted as dashed lines. However, an important point should be noted. For the calculations of the JKR dependencies with Equations (2) and (3), we employed not the actual parameters but effective parameters, as listed in Table 1.

The Poisson ratio was the same, ν=0.47, for all calculations. The primary reason for using effective (fitted) values of elastic modulus E was the limited thickness of the elastomer, which does not permit the direct application of solutions derived for the half-space limit in the JKR theory. As demonstrated experimentally in our previous work [35], even if the elastomer thickness is three times larger than the contact diameter, the solution of the contact problem using half-space approximation results in a contact stiffness that is reduced by more than 20%. This implies that the experimental results will exhibit higher normal forces at a large indentation depth. This discrepancy arises because, as the indentation depth increases, the system deviates from the conditions assumed in the half-space limit, where the JKR theory is valid. Consequently, higher values of elastic modulus were used in the calculations to achieve numerical agreement with experimental data.

It follows from Figure 5 that for the smallest angle Φ=10°, the experimental data show a much higher pull-off force (adhesive strength) than the proposed JKR curve. These curves do resemble a different JKR dependence.

In the case of the smallest cylinder ($R_{3}\approx 15\;\mathrm{mm}$) with an angle of 10°, upon reaching the maximum indentation depth, the graph declines monotonically until it reaches the pull-off force, which in contrast to the other experiments is at a positive indentation depth. Subsequently, the force increases, though not for an extended period. Such behavior is usually caused by the influence of chemical inhomogeneities on the contacting surfaces, surface roughness [36], or the presence of third-body particles (contaminants, wear particles) or other geometrical irregularities in the contact zone or even inside the elastomer [37,38,39]. All these factors can lead to the formation of additional stable contact configurations, which manifest as non-monotonicities in the F(d) dependencies.

**Figure 6:**
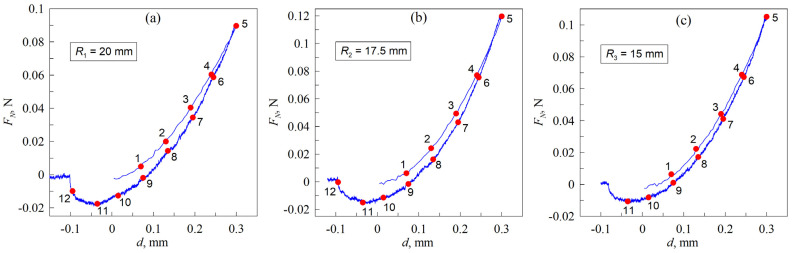
Dependencies of the normal force $F_{N}$ vs. indentation depth d for different contact configurations, where $R_{1}\approx 20\;\mathrm{mm}$ (**a**), $R_{2}\approx 17.5\;\mathrm{mm}$ (**b**), and $R_{3}\approx 15\;\mathrm{mm}$ (**c**). The experimental series was taken for an angle of Φ=50°. The red points on the graph illustrate the reading points where the contact area was evaluated.

Figure 6 shows the dependencies $F_{N}\left(d\right)$ in full indentation–detachment cycles for all three cases at a fixed angle between the axes of cylinders Φ. For the purposes of analysis, a case with the angle of Φ=50° was selected for investigation. Each dependence contains twelve reading points. As a consequence of the hysteresis behavior exhibited by the normal force with respect to the indentation depth, the value of the normal force in the indentation phase and the detachment phase do not coincide at the same indentation depth. As previously discussed in [34], the specific work of adhesion $\gamma_{12}$ is observed to be significantly greater in the detachment phase than in the indentation phase, which is a typical behavior for adhesive contacts [40]. Consequently, the augmented adhesive force, which is oriented in opposition to the elastic reaction force of the elastomer, reduces the resultant force at that depth. Figure 6 illustrates that the dependencies $F_{N}\left(d\right)$ are relatively smooth during the indentation phase. However, following the change in direction, the line is overlapped by a noise signal with a very small amplitude. Such an effect is caused by two aspects. First, the indentation was performed at a higher speed, and therefore, the dependence $F_{N}\left(d\right)$ corresponding to the indentation contains fewer points. Second, the jittering in the dependence could be attributed to the retraction of the contact area, which does not proceed smoothly due to factors such as surface roughness, material inhomogeneities, or the presence of third-body particles.

**Figure 7:**
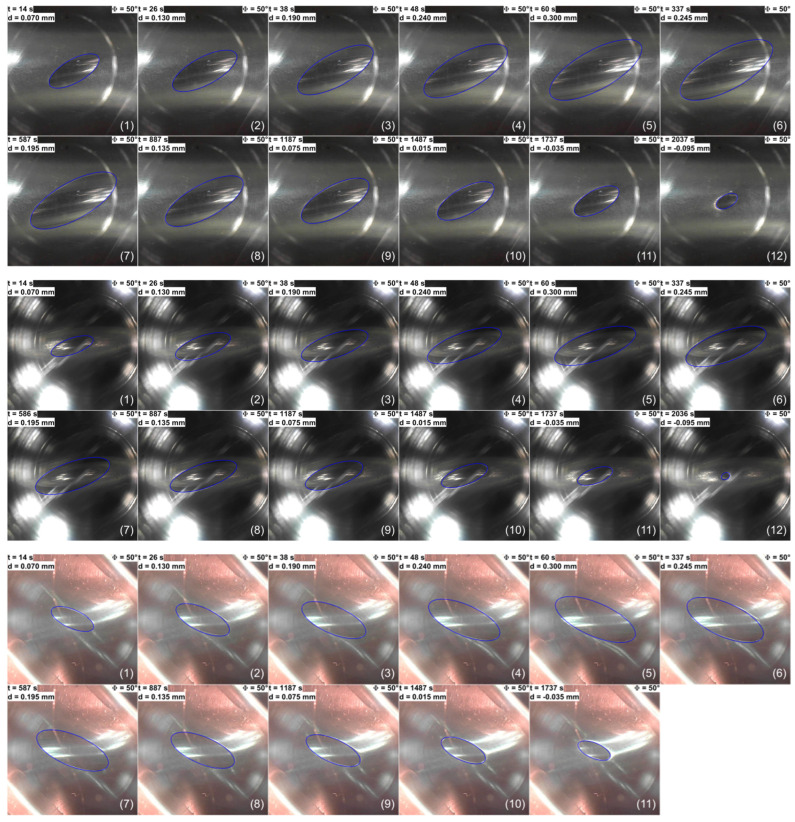
Photographs of the contact area for the three different contact configurations: upper group of pictures corresponds to the case $R_{1}\approx 20\;\mathrm{mm}$; for the middle group, $R_{2}\approx 17.5\;\mathrm{mm}$; and for the bottom group, $R_{3}\approx 15\;\mathrm{mm}$. The rotation angle for all groups of pictures was held constant at Φ=50°. The indenter was first indented and then detached from the lower elastomer sheet. The numbered contact area pictures match the points on the dependencies depicted in Figure 6.

The same dependencies as for the biggest half-cylinder $R_{1}$ can be observed in the contact pair for the hollow cylinder with the next smallest radius of $R_{2}\approx 17.5\;$mm. The most notable distinction is that at the final instance of detachment observed in the second experiment, the contact area is markedly smaller and closely approximates the shape of a circle (see Figure 7). It should be noted that in all figures showing contact configurations (e.g., Figure 7), the boundary line of the contact is depicted as a solid, closed blue line. This was decided because the steel indenter used in all experiments was polished to reduce surface roughness. Under such conditions, the difference between the elastomer in contact and out of contact is not clearly distinguishable. However, in the dynamic Supplementary Videos (Videos S1–S3), the contact area is unambiguously visible. For the production of the videos, we used the original images of the contact area without adding the additional contact boundary line.

In the case of the smallest half-cylinder $R_{3}$, only 11 points were selected, given that the indenter completely detaches from the elastomer at smaller negative indentation depths. It should be noted, however, that the maximum adhesion force is observed at the same indentation depth for all contact configurations. The associated normal force at that particular depth is nearly identical for the two largest substrates, at approximately $F_{N}\approx -0.016\;N$. In contrast, the force is smaller for the smallest cylinder, at only $F_{N}\approx -0.01\;N$, despite being at the same depth. However, compared to the largest half-cylinders, $R_{3}\approx 15\;\mathrm{mm}$ exhibits a higher normal force at the maximal indentation depth of $d_{max}=0.3\;\mathrm{mm}$ (see also Figure 4).

**Figure 8:**
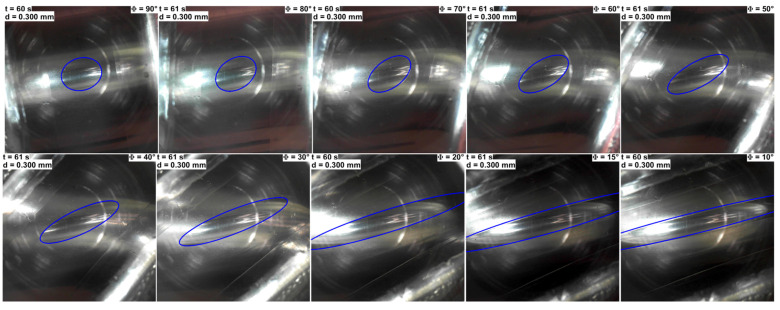

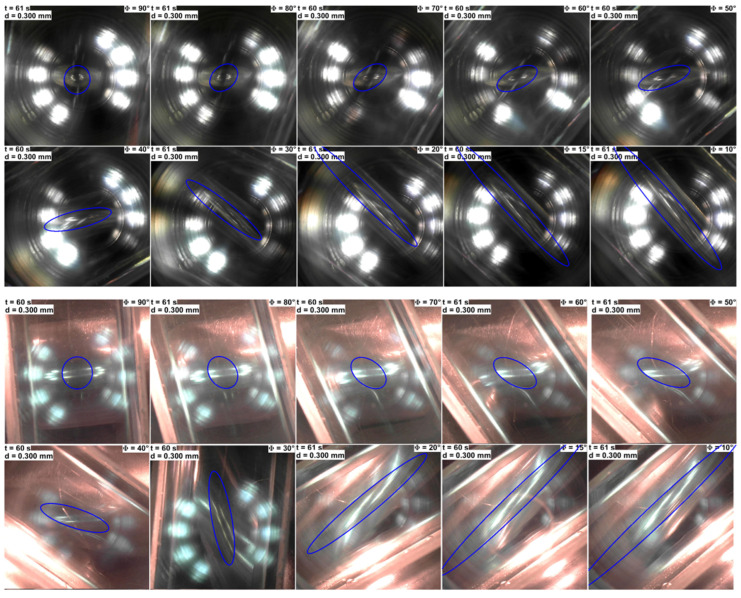
Photographs of the contact area for the three different contact configurations (upper group of pictures corresponds to the case $R_{1}\approx 20\;\mathrm{mm}$; for the middle group, $R_{2}\approx 17.5\;\mathrm{mm}$; and for the lower group, $R_{3}\approx 15\;\mathrm{mm}$) at different angles. The indentation depth for all pictures is the same, $d=d_{max}=0.3$ mm.

Additionally, the case was considered, where the indentation depth was maintained at the maximal value $d_{max}=0.3\;\mathrm{mm}$ and the contact area was observed at ten different angles, ranging from 90° to 10°. It should be noted that the photographs of the contact area were taken from different experimental series. In each series, the indenter is first fully indented and subsequently fully detached from the elastomer surface. As anticipated, at the angle of 90°, where the axes of the contacting bodies are perpendicular to each other, the contact area is observed to be nearly perfectly circular. As illustrated in Figure 8, the contact area undergoes a transformation from a circular to an elliptical shape as the angle decreases. Up until the angle of 50°, the changes are minimal and the eccentricity of the contact area only exhibits a slight increase. At an angle of 40°, the characteristics of an ellipse become more pronounced, and the ratio between the minor and major axes increases rapidly. As illustrated in the photographs in Figure 8, the tips at the major axis of the contact area exhibit a greater degree of pointedness. Additionally, the area of contact increases. Nevertheless, the most notable increase is observed in the value of the major axis, reaching a point where the viewing area is not sufficiently large to encompass the entire contact area. As the angles approach 0°, where the two cylinders are perfectly parallel to each other, and the contact should be a rectangle with the length of the steel cylinder, the major axis continues to increase. However, it can be observed that the width of the ellipse does not change significantly, compared to the length. These observations are consistent across all contact pairings.

Note that for the middle-size substrate, the contact area appears to be smaller than the other two, as it can be observed in its entirety within the viewing window. This occurs because, at larger indentation depths, not only the elastomer but also the underlying cylinders undergoes deformation, and in all cases, the cylinders have different properties (filled half-cylinder made from rubber, $R_{1}$; hollow, $R_{2}$; and filled half-cylinders made from acrylic glass, $R_{3}$). This was discussed earlier in the description of Figure 3.

**Figure 9:**
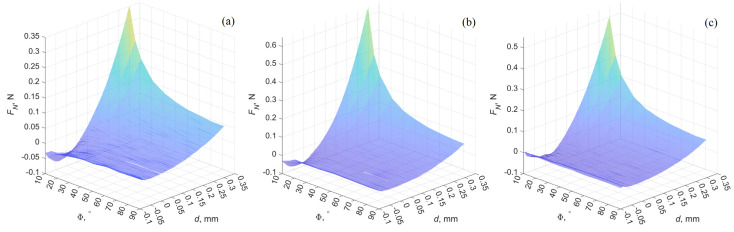
Three-dimensional plots of the three different contact configurations during the detachment phase, illustrating the normal force $F_{N}$ as a function of the rotation angle Φ and the indentation depth d. Plots present the results of experiments conducted with cylinders $R_{1}\approx 20$ mm (**a**), $R_{2}\approx 17.5\;$mm (**b**), and $R_{3}\approx 15\;$mm (**c**).

In Figure 9, the 3D dependencies of the normal force $F_{N}$ on the indentation depth d and the rotation angle Φ are depicted for the detachment phase. These dependencies are similar to those presented in Figure 3, with the distinction that Figure 3 shows cross-sections of 3D dependencies at fixed angles of Φ, whereas Figure 9 presents the full 3D dependencies $F_{N}\left(\Phi ,d\right)$. Representing force dependencies in 3D form has advantages, as it allows for visualizing both the influence of indentation depth d at a fixed rotation angle Φ and the influence of the angle Φ at a fixed indentation depth d.

The Supplementary Video S1 for the largest half-cylinder demonstrates that during the indentation phase, the contact border is a “clean” thin line. However, during the subsequent detachment phase, the border exhibits increased thickness, irregularity, and blurriness, suggesting a less defined and more diffuse boundary. As the angle between the cylinders decreases and they become parallel, the initial contact area increases to a point where it is no longer fully captured by the camera. Furthermore, in the experimental series where the upper cylinder is first indented and then detached, a substantial part of the contact area undergoes a more pronounced transformation during the detachment phase. During the indentation phase, it is evident that the elliptical shape of the contact area is not symmetrical. It can be observed in the Supplementary Videos that the lower part of the contact area is thicker than the upper part. This phenomenon is more pronounced in experiments conducted with higher angles. As the rotation angle decreases, the discrepancy between the indentation and detachment curves in the $F_{N}\left(d\right)$ dependence increases. This can be attributed to the expansion of the contact area, necessitating a greater amount of applied force to separate the two bodies.

The same observations can be made with regard to the contact pair of the hollow half cylinder $R_{2}\approx 17.5\;$mm with the steel indenter. In contrast to the aforementioned cylinder, at small rotation angles the contact diminishes more slowly at the major axis, resulting in a very long and thin contact area. The contact area is particularly challenging to discern, especially at modest rotation angles. This phenomenon may be attributed to the presence of a hollow cylinder, which gives rise to the formation of white streaks on the surface, potentially overlapping with the border of the contact area.

The final experimental series, which used the smallest half cylinder with an approximate radius of $R_{3}\approx 15\;$mm, also demonstrated that the contact borders are more evident during the detachment phase. This is, however, only advantageous in the initial experiments, as with smaller rotation angles, the borders are blurred and extremely challenging to detect. It is also noteworthy that the deformation of the elastomer substrate is easily observed, or in fact is much more visible than in the other experimental series. This phenomenon can be attributed to the heightened curvature of the elastomer sheet, which generates a greater tensile force at the upper surface. Moreover, the contact area does not resemble an ellipse at shallow indentation depths during the detachment phase. Instead of an ellipse, it resembles a rectangle with rounded tips at the ends of the major axis. The tapering of the contact area along the major axis is no longer evident.

## 4. Discussion

The white borders in the contact area pictures and discrepancies in normal force between indentation and detachment can be explained by the presence of an adhesive neck, which can only be observed during the detachment phase [28].

**Figure 10:**
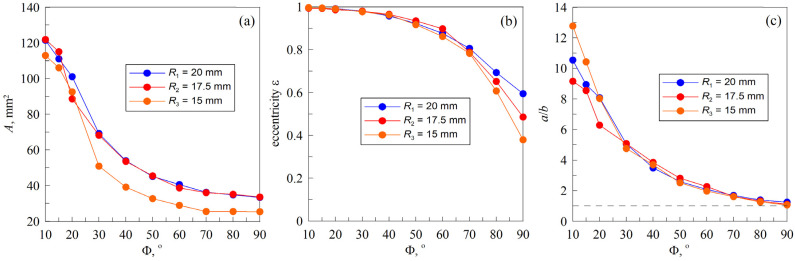
Dependencies of the contact area A (**a**), eccentricity ε (**b**), and the ratio between major and minor half-axes a/b (**c**) vs. angle Φ at maximal indentation depth $d=d_{max}=0.3$ mm. Eccentricity $\varepsilon =\sqrt{1-b^{2}/a^{2}}$ is the eccentricity of the analytical ellipse that recreate the real contact ellipse.

In Figure 7 and Figure 8, the original contact area is outlined in blue, which represents an analytical ellipse. To calculate the parameters of the ellipse, the positions of the endpoints of the minor and major axes were manually determined. Based on these data points, the length and width of the ellipse were calculated and subsequently plotted on the photograph. It is probable that errors have been made in detecting the tips of the minor axis, as they are not as prominent as the tips of the major axis and there is a likely asymmetric detachment of the indenter from the substrate. Consequently, the analytical line deviates from the contact border in certain figure panels. Moreover, for small angles where the contact area exceeds the camera frame, the endpoints of the major axis can only be predicted. As illustrated in Figure 10, a reduction in the angle Φ results in an increase in the contact area, accompanied by an increase in the eccentricity to a value of 1, which indicates that the elliptical contact area becomes strongly elongated.

It should be noted that the endpoints of the minor and major axes for $R_{2}\approx 17.5\;\mathrm{mm}$ were particularly challenging to discern. It was anticipated that an increase in substrate radius would result in a proportional increase in contact area. This can be seen in the discrepancy between the lines of $R_{2}\approx 17.5\;\mathrm{mm}$ and $R_{3}\approx 15\;\mathrm{mm}$. (Note that as the pixel to length ratio was not recorded for $R_{1}\approx 20\;\mathrm{mm}$, the same pixel-to-length ratio as for $R_{3}\approx \;15\;\mathrm{mm}$ was chosen, as both half cylinders were fully filled.) However, as can be seen in Figure 10, for some reason, the dependency of the contact area for $R_{1}\approx 20\;\mathrm{mm}$ and $R_{2}\approx 17.5\;\mathrm{mm}$ displays a similar trend, whereby the contact area is larger for smaller angles, but then decreases rapidly until an angle of 40° is reached, whereupon the decrease becomes very gradual. All experimental series exhibit a similar pattern with regard to the variation in eccentricity ε. It should be noted that, in all cases, the contact area at 90° deviates from a circular shape (see also Figure 8), resulting in an eccentricity ε that does not approach zero, as shown in Figure 10b. For the same reason, the ratio a/b at an angle of 90° is slightly greater than one (see Figure 10c).

From Figure 10, it can be seen that the ratio between the major and minor axis changes with the rotation angle, but for all experimental series, the dependency follows the same trend from an angle of 30° onwards. The discrepancies observed at lower angles can be attributed to the difficulties in accurately determining the precise position of the axis ends.

**Figure 11:**
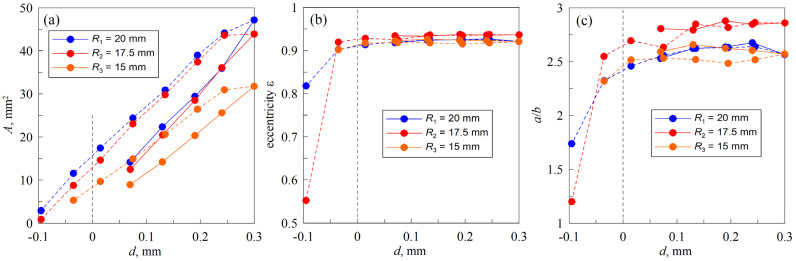
Dependencies of the contact area A (**a**), eccentricity ε (**b**), and the ratio between major and minor half-axes a/b (**c**) vs. indentation depth d at fixed angle Φ=50°. Eccentricity $\varepsilon =\sqrt{1-b^{2}/a^{2}}$ is the eccentricity of the analytical ellipse that recreates the real contact ellipse.

In contrast to Figure 10, Figure 11 depicts the dependencies at varying indentation depths at a constant angle of Φ=50°.

Overall, it can be seen that the eccentricity stays nearly constant over all indentation depths. Nevertheless, the ratio between the two axes demonstrates a more pronounced discrepancy, especially for the smallest half-cylinder. Since Figure 11 presents the dependencies for the complete indentation–detachment cycle, hysteresis of the contact area is clearly visible here (see Figure 11a). For a better visualization of the hysteresis, points corresponding to the indentation are connected with solid lines, while the dependencies for the detachment are shown with dashed lines. Interestingly, the other two panels of the figure, which demonstrate eccentricity ε and the ratio a/b between the half-axes of the ellipse, do not exhibit hysteresis. This indicates that both during indentation (increasing the contact area) and detachment (decreasing the contact area), the ratio between the axes of the ellipse remains approximately the same. Thus, the ratio between the axes of the ellipse a/b depends only on the angle Φ between the contacting cylinders and does not depend on the loading history. At the same time, the size of the ellipse (contact area A) does depend on the loading history, as demonstrated in Figure 11a, where this is shown as a clearly visible hysteresis.

## 5. Conclusions

This work describes a series of experiments on the indentation of a smooth steel cylinder into a cylindrical elastomer. The experiments followed an indentation–detachment scheme with varying angles between the axes of the cylinders. At all angles between the cylinder axes, the contact area took the shape of an ellipse. It was shown that as the angle between the cylinder axes decreased and the contact ellipse elongated, there was an increase in the contact area, contact stiffness, and adhesive force. The hysteresis of force and contact area, arising during the complete indentation–detachment cycle, was analyzed. It was demonstrated that for a fixed angle between the contacting cylinders, the eccentricity of the ellipse (or the ratio of its axes) did not depend on the loading history (e.g., the depth of indentation).

In future works, we plan to investigate the effect of tangential shear on the shape of the contact. In adhesive tangential contacts, as has been repeatedly observed experimentally for spherical indenters, the contact boundary at the front and rear edges of the contact area evolves differently. This results in a loss of symmetry in the contact patch. It is hypothesized that in the case of elliptical contact, tangential shear should also lead to a symmetry loss in the contact and cause the contact area to deviate from an elliptical shape, or at least alter the geometric parameters of the elliptical region.

## Figures and Tables

**Table 1:** The effective parameters of the elastomer used to calculate the JKR curves shown in Figure 5.

Bottom Cylinder Radius *R_i_* (mm)	Elastic Modulus *E_eff_* (kPa)	Specific Work of Adhesion $\gamma_{12}\;(J/m^{2})$
20	90	0.3
17.5	110	0.35
15	100	0.25

## Data Availability

The original contributions presented in the study are included in the article/Supplementary Materials; further inquiries can be directed to the corresponding author.

## Supplementary Materials

The following supporting information can be downloaded at: https://www.mdpi.com/article/10.3390/ma18020375/s1, Video S1: Experimental series, where the lower half-cylinder with the overlaying elastomer has a radius of $R_{1}\approx 20\;\mathrm{mm}$. The steel indenter is indented with a velocity of v=5 µm/s; upon reaching the maximal indentation depth of $d_{max}=0.3\;\mathrm{mm}$, the indenter is pulled out of the elastomer with a much slower velocity of v=0.2 µm/s until complete detachment. The experiment is repeated for 10 different angles (Φ=90°, 80°, 70°, 60°, 50°, 40°, 30°, 20°, 15°, 10°). The left panel shows the recorded contact area, whereas the right panel depicts the force–depth $F_{N}\left(d\right)$ dependency for all angles in different colors; Video S2: It is similar to Video S1, but with the difference that the lower substrate has a radius of $R_{2}\approx 17.5\;\mathrm{mm}$; Video S3: It is similar to Video 1, but with the difference that the lower substrate has a radius of $R_{3}\approx 15\;\mathrm{mm}$.
